# Adjuvant treatment for high‐risk salivary gland malignancies and prognostic stratification based on a 20‐year single institution experience

**DOI:** 10.1002/hsr2.195

**Published:** 2020-10-07

**Authors:** Benjamin E. Onderdonk, Everett E. Vokes, Michael Gwede, Elizabeth Blair, Nishant Agrawal, Daniel J. Haraf

**Affiliations:** ^1^ Department of Radiation and Cellular Oncology University of Chicago Chicago Illinois; ^2^ Department of Medicine, Section of Hematology/Oncology University of Chicago Chicago Illinois; ^3^ Pritzker School of Medicine University of Chicago Chicago Illinois; ^4^ Department of Otolaryngology University of Chicago Chicago Illinois

**Keywords:** chemoradiation, chemotherapy, radiation, risk factors, salivary gland

## Abstract

**Background and Aim:**

Retrospective analysis of the utility of adjuvant radiation (RT) or chemoradiation (CRT) and identify prognostic features for patients with high‐risk head and neck salivary gland cancers.

**Methods:**

From 1/1997 to 12/2017, 108 patients underwent surgery, and RT (n = 50) or CRT (n = 58) for positive lymph node(s), extracapsular extension, perineural invasion, lymphovascular space invasion, positive/close margin, and/or grade 3 disease. Outcomes were estimated with the Kaplan‐Meier method. Significant predictors identified through regression analyses were incorporated into multivariable regression (MVA). Toxicities were compared using chi‐square.

**Results:**

The median follow‐up was 52 months (range: 3‐226). The number of risk factors (RFs) between RT and CRT groups were: 0 to 1 (44% vs 7%), 2 to 3 (48% vs 41%), or 4 to 6 (8% vs 52%), respectively (*P* < .01). On MVA, stage 3 or 4 disease predicted worse outcomes including overall survival (HR 4.55, *P* = .01). Increasing number of RFs predicted worse disease‐free survival, distant metastasis‐free survival, and overall survival (2‐3 RFs: HR 3.38, *P* = .03; 4‐6 RFs: HR 5.78, *P* < .01), but not locoregional control (*P* = .54). So, adjuvant CRT may have provided comparable locoregional control for patients with more adverse features, but the CRT did not translate into improved distant control. There was no difference in acute or late grade 3+ toxicities, or parenteral nutrition (*P* = .98, *P* = .85, and *P* = .83), respectively.

**Conclusions:**

Adjuvant CRT provides adequate locoregional control in patients with more adverse RFs. The absolute number of RFs serves prognostic significance and should be considered in future prospective trials.

## 1 INTRODUCTION

Salivary gland malignancies represent a rare type of cancer. Treatment is based on retrospective data given the relatively small number of affected patients and paucity of randomized trials to guide treatment decisions. Surgery is considered the mainstay of treatment. However, several high‐risk features have been identified such as: perineural involvement (PNI), lymphovascular space invasion (LVSI), positive/close (<2 mm) margin, grade 3 disease, extracapsular extension (ECE), or positive lymph nodes (+LN).

Adjuvant radiation has been used with success to improve locoregional control in high‐risk disease,^1^, ^2^, ^3^ and even overall survival in those with ECE and/or positive margin.^4^ However, the addition of chemotherapy, used in good performance patients, has been met with mixed results and with few drugs reporting single agent activity.^5^, ^6^, ^7^, ^8^ Given these results, it remains possible that some drugs may still work as radiation sensitizers.

While large prospective randomized trials continue to accrue (NCT01220583, NCT02998385) or report (NCT01488838), there remain limited comparisons between adjuvant chemoradiation (CRT) to radiation therapy (RT) alone in patients with high‐risk salivary gland cancer. Moreover, the prognostic value of different high‐risk features, or a combination of which remains poorly understood. While we wait for this large prospective evidence, we investigate the utility of adjuvant CRT compared to RT alone and identified prognostic features from our 20‐year institutional experience.

## 2 METHODS

### 2.1 Patient characteristics

Patients with histologically confirmed high‐risk head and neck salivary gland carcinomas were retrospectively identified through the electronic medical record. All patients signed the institutional review board (IRB17‐0489) protocol consent. Those identified included those that received definitive surgical resection followed by adjuvant RT alone or CRT. Patients who previously received RT to the head and neck were excluded from data collection. Pre‐treatment evaluation included assessing pathologic specimens, and operative findings. The risk factors (RFs) included: +LN, ECE, PNI, LVSI, positive/close margin, and grade 3 disease. These RFs were summed to reach the number of factors (ranging from 0 to 6).

Patients had to have a confirmation of localized disease through head and neck computerized tomography (CT) and/or magnetic resonance imaging (MRI) and at least CT chest to rule out metastatic disease. Disease stages were coded according to the AJCC 7th edition.

### 2.2 Treatment

The adjuvant RT cohort received 2 Gy daily fractions. The adjuvant CRT utilized most frequently comes from a previously reported practice of 4 to 6 alternating “cycles” of weekly TFH or FH with RT.^9^, ^10^, ^11^ This regimen constitutes: infusion 5‐fluorouracil (600 mg/m^2^/d × 5 days), hydroxyurea (500 mg PO twice‐daily [BID]), with/without 5 days of paclitaxel (100 mg/m^2^ on day 1), and either 1.8 to 2 Gy daily (QD) RT or 1.5 Gy BID RT from days 1 to 5. The cycle of CRT is then followed by a 9‐day break, after which the next cycle of CRT begins. The regimen used varied over the years as treatment protocols evolved in an effort to improve efficacy. While earlier patients were treated with FH and QD fraction RT, more recent patients received TFH and either QD or BID RT.^8^, ^9^, ^10^

### 2.3 Endpoints

Follow‐up data was gathered from the notes in the electronic medical record, which included: radiation oncology, otolaryngology, medical oncology, telephone encounters, and event notes. Head and neck acute and late toxicities were assessed with the use of RTOG toxicity scales. Acute toxicities were defined as those occurring within 90 days of treatment, whereas late toxicities were defined as those occurring after 90 days of treatment. Due to the lack of laboratory reports in early versions of the electronic medical record, hematologic toxicities were not recorded. The use of artificial feeding tubes or total parenteral nutrition (TPN) was documented when the use of these methods for supplemental nutrition occurred as a result of RT or CRT.

Locoregional control was defined from the time of surgery until evidence of recurrence within the primary tumor bed, adjacent to the tumor bed, or within the regional lymphatics. Distant metastasis‐free survival was defined from the time of surgery until evidence of recurrence outside of the head and neck region. Disease‐free survival was defined from the time of surgery until recurrence or death. Overall survival was defined from the time of surgery until death from all causes.

### 2.4 Statistical methods

The survival outcomes were estimated using the Kaplan‐Meier method and compared using the log‐rank test. Multivariable Cox proportional hazards model (MVA) was performed for any significant factors on univariate analysis. From this initial model, backward stepwise regression was then performed to remove factors that were not significant, and to find the most parsimonious model that minimized Akaike Information Criteria and Bayesian Information Criterion. RTOG toxicity tables were used to score the maximum acute and late head and neck toxicities and compared using the chi‐square test.

## 3 RESULTS

From January 1997 to December 2017, 108 patients were treated with surgery, followed by adjuvant RT alone (n = 50) or CRT (n = 58) for high‐risk pathologic features. The median follow‐up duration was 52 months (range: 3‐226). There were a total of 11 different histologies, with the most common being: adenoid cystic carcinoma (n = 30), followed by mucoepidermoid (n = 20), and salivary duct adenocarcinoma (n = 18). The most salivary gland location was the parotid (n = 73). Twenty‐two percent were in minor salivary gland locations. The rest of patient characteristics are shown in Table 1.

**Table 1:** Baseline patient characteristics

Patient characteristic	Adjuvant RT N = 50 (46%)	Adjuvant CRT N = 58 (54%)	
Male gender	16 (32%)	34 (59%)	*P* = .01
Average age (range) years	56 (16‐90)	59 (21‐85)	*P* = .13
*Primary site*			*P* = .33
Parotid	31 (62)	42 (72)	
Submandibular	7 (14)	4 (7)	
Sublingual	0	1 (2)	
Minor—Hard palate	7 (14)	4 (7)	
Minor—Nasopharynx	1 (2)	2 (3)	
Minor—Base of tongue	0	2 (3)	
Minor—Other location	4 (8)	3 (5)	
*Histology*			*P* = .24
Adenoid cystic carcinoma	18 (36)	12 (21)	
Mucoepidermoid Carcinoma	10 (20)	10 (17)	
Salivary duct adenocarcinoma	5 (10)	13 (22)	
Carcinoma Ex‐pleomorphic adenoma	4 (8)	9 (16)	
Acinic cell	6 (12)	4 (7)	
Squamous cell	4 (8)	6 (10)	
Myoepithelial	1 (2)	0	
Other (oncocytic, clear cell, MASC, LELC)	2 (4)	4 (7)	
*T‐Stage*			*P* < .01
1	16 (32)	7 (12)	
2	22 (44)	13 (22)	
3	8 (16)	20 (34)	
4a	4 (8)	18 (31)	
*N‐Stage*			
0	45 (90)	24 (41)	*P* < .01
1	3 (6)	8 (14)	
2b	2 (4)	24 (41)	
2c	0	2 (3)	
*Stage*			*P* < .01
I	15 (30)	2 (3)	
II	19 (38)	5 (9)	
III	10 (20)	14 (24)	
IVa	6 (12)	37 (64)	
Positive/close margin	40 (80)	47 (81)	*P* = 1.0
Extracapsular/nodal extension	1 (2)	21 (36)	*P* < .01
Perineural invasion	18 (36)	35 (60)	*P* = .01
Lymphovascular space invasion	6 (12)	24 (41)	*P* < .01
Grade 3	21(42)	46 (79)	*P* < .01
Number of risk factors (LN, ECE, PNI, LVI, +/close Margin, Gr 3: range 0‐6)	0‐3 (6)	0‐0	*P* < .01
	1‐19 (38)	1‐4 (7)	
	2‐16 (32)	2‐13 (22)	
	3‐8 (16)	3–11 (19)	
	4‐4 (8)	4‐13 (22)	
	5‐0	5‐10 (17)	
	6‐0	6‐7 (12)	

Abbreviations: ECE, extracapsular extension; PNI, perineural involvement.

### 3.1 Treatment characteristics

The median dose of RT was 66 Gy (range: 50‐74 vs 43.2‐70, *P* = .15) in the adjuvant RT and CRT groups, respectively. In the CRT group, 50% received BID treatments. Both 3D conformal radiation therapies (6.9% vs 16%) and intensity‐modulated radiotherapy (IMRT) (84% vs 93.1%, *P* = .22) techniques were utilized in the RT alone and CRT groups, respectively.

In the CRT group, the majority of chemotherapy regimens consisted of TFH (79%), followed by FH (17%), then weekly cisplatin (2%), and unknown/other (2%). Only three patients received induction carboplatin/paclitaxel followed by CRT.

### 3.2 Outcomes

The unadjusted 5‐year locoregional control estimates were (92% vs 82%, *P* = .05, Figure S1), 5‐year disease‐free survival estimates were (72% vs 42%, *P* = .02, Figure S2), 5‐year distant metastasis‐free survival estimates were (76% vs 49%, *P* = .02, Figure S3), and the 5‐year overall survival estimates were (94% vs 64, *P* < .01, Figure S4) between the RT alone and CRT groups, respectively. However on MVA, CRT was no different than RT alone for locoregional control (*P* = .57), disease‐free survival (*P* = .37), distant metastasis‐free survival (*P* = .80), or overall survival (*P* = .42).

On MVA, locoregional control was only associated with stage 3 or 4 disease compared to stage 1 or 2 (HR: 2.67, *P* < .01). Figure S5 depicts the 5‐year locoregional control for stage 1 (100%), stage II (100%), stage III (65%), and stage IVa (89%). Stage 3 or 4 disease was also significant for disease‐free survival, distant metastasis‐free survival, and overall survival (HR 4.67, *P* = .02).

**Figure 1:**
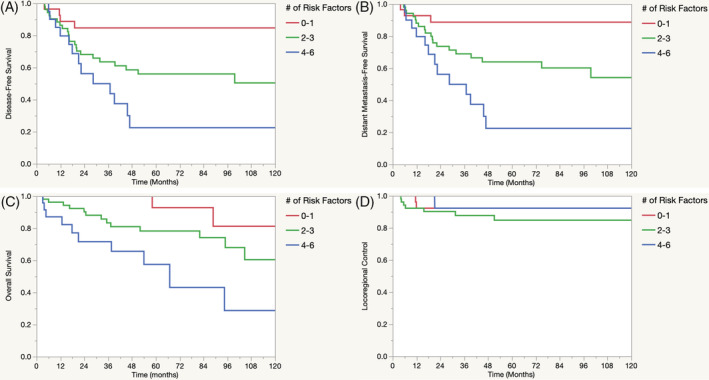
A‐D, DFS, DMFS, OS, and LRC by the number of risk factors. Kaplan‐Meier survival estimates for DFS, DMFS, OS, and LRC by the number of risk factors present. Risk factors included: PNI, LVSI, positive/close (<2 mm) margin, grade 3 disease, ECE, or positive lymph nodes (+LN). DFS, disease‐free survival; DMFS, distant metastasis‐free survival; ECE, extracapsular extension; HR, hazard ratio; LRC, locoregional control; LVSI, lymphovascular space invasion; MVA, multivariable analysis; OS, overall survival; PNI, perineural involvement

In addition to stage, the total number of RFs was associated with disease‐free survival (2‐3 RFs HR 3.04, *P* = .03; 4‐6 RFs HR 3.98 *P* = .02, Figure 1A), distant metastasis‐free survival (2‐3 RFs HR 3.38, *P* = .03; 4‐6 RFs HR 5.78, *P* < .01, Figure 1B), and overall survival (2‐3 RFs HR 3.84, *P* = .04; 4‐6 RFs HR 5.42 *P* = .02, Figure 1C). As a comparison, Figure 1D depicts locoregional control by number of RFs, which remained non‐significant (*P* = .54).

Of note, imbalances in therapies existed: 87% of patients with lymph node positivity (*P* < .01), and 95% of patients with ECE (*P* < .01) were treated with adjuvant CRT, thus comparison with the RT alone group was not feasible. Figures S6 and S7 depict OS as a function of +LN (*P* = .16) and ECE (*P* < .01), respectively. The MVA and model outputs are shown in Table 2.

**Table 2:** Multivariable analysis for survival outcomes

Variable	LRC (HR, *P* value)	DFS (HR, *P* value)	DMFS (HR, *P* value)	OS (HR, *P* value)
CRT (+/−)	1.54, *P* = .57	0.78, *P* = .37	0.88, *P* = .80	1.59, *P* = .42
Stage (3/4 vs 1/2)	**2.67, *P* < .01**	**3.91, *P* < .01**	**2.80, *P* = .04**	**4.67, *P* = .02**
# of RF: 2–3 vs 0‐1; 4‐6 vs 0‐1; 4‐6 vs 2‐3	‐	**3.04, *P* = .03**	**3.38, *P* = .03**	**3.84, *P* = .04**
		**3.98, *P* = .02**	**5.78, *P* < .01**	**5.42, *P* = .02**
		1.31, *P* = .49	1.71, *P* = .19	1.41, *P* = .45
Overall *F* test of entire model	***P* < .01**	***P* < .01**	***P* < .01**	***P* < .01**

*Note*: Bold *P* values indicate statistical significance or trend.

Abbreviations: CRT, chemoradiation; DFS, disease‐free survival; DMFS, distant metastasis‐free survival; HR, hazard ratio; LRC, locoregional control; OS, overall survival; RF, risk factors (lymph node positivity, extranodal extension, lymphovascular space invasion, perineural invasion, positive/close surgical margin, grade 3).

### 3.3 Toxicity

Overall, there were no differences in RTOG head and neck acute grade 3+ toxicities between the adjuvant RT alone group (36.0%) compared to the adjuvant CRT group (36.2%; *P* = .98). There was one acute grade 5 toxicity in the RT alone group, which occurred in a patient who succumbed to a ventricular arrhythmia due to the inability to take amiodarone secondary to acute mucositis. Furthermore, there were no differences in RTOG head and neck late grade 3+ toxicities (6.0% vs 6.9%; *P* = .85), or rates of G‐tube/TPN use (8.0% vs 6.9%; *P* = .83). When comparing those that received 3D conformal RT to IMRT, there were no differences in acute toxicities (33.33% vs 36.5%; *P* = .83), late toxicities (8.3% vs 6.3%; *P* = .79), or G‐tube/TPN rates (0.0% vs 8.3%; *P* = .16). A tabular form of the toxicities is shown in Table 3.

**Table 3:** Head and neck toxicities

Maximum RTOG toxicity	H&N acute Grade 3+ toxicity (%)	H&N late Grade 3+ toxicity (%)	G‐Tube/TPN (%)
RT Alone	36.0%^a^	6.0%	8.0%
CRT	36.21%	6.9%	6.9%
Chi‐square	*P* = .98	*P* = .85	*P* = .83
3DCRT	33.33%	8.33%	0%
IMRT	36.46%	6.25%	8.33%
Chi‐square	*P* = .83	*P* = .79	*P* = .16

a There was one acute grade 5 toxicity in the RT alone group.

Abbreviations: 3DCRT, 3D conformal radiation therapy; CRT, chemoradiation; G‐tube, gastrostomy tube; H&N, head and neck; IMRT, intensity modulated radiation therapy; RT, radiation therapy; RTOG, radiation therapy oncology group; TPN, total parenteral nutrition.

## 4 DISCUSSION

Unlike in high‐risk head and neck squamous cell carcinomas, where adjuvant chemoradiation prospectively demonstrates an improvement in locoregional control and overall survival in the,^12^ the benefit in high‐risk salivary gland malignancies largely remains unknown. While practitioners await prospective results for a definitive answer, they are currently guided by small retrospective CRT studies,^5^, ^6^ or from large national cancer database analyses.^7^ However, there remain limitations with the former having fewer patients receiving adjuvant CRT, and inherent biases within large national databases taint the comparisons between CRT and RT.

With these limitations in mind, this study represents the largest single‐institution use of adjuvant CRT for high‐risk salivary malignancies, and utilizes a comparison RT alone group. Although there were more high‐risk features (including nodal disease and ECE) in the CRT group compared with the RT alone group (Table 1), this study demonstrated that comparable survival outcomes between the groups when controlling for these other variables.

In addition to previous reports for nodal stage,^13^ overall advanced stage of disease in our report remains a poor prognostic factor. Of note, for the first time, the number of pathologic RFs was associated with worse disease‐free survival, distant metastasis‐free survival, and overall survival, but not locoregional control. The discrepancy in outcomes is notable since the CRT group had more adverse features (4‐6 RFs: 52% vs 8%, *P* < .01).

The central implication of this mismatch is that the use of CRT may have compensated for high‐risk features in the treatment group, resulting in comparable locoregional control. Thus, the use of adjuvant CRT may be considered for patients with four or more high‐risk features to provide comparable locoregional control. Importantly, while 5‐year locoregional control may be comparable (92%, Figure 1D) for patients with a significant amount of adverse features, the use of CRT did not translate into an improvement in 5‐year disease‐free survival (22%, Figure 1A), 5‐year distant metastatic‐free survival (22%, Figure 1B), or 5‐year overall survival (57%, Figure 1C).

Although these findings are notable, this study has inherent limitations due to its retrospective nature. Also, the discrepancy in nodal disease between groups limited our ability to individually compare CRT vs RT alone in this subset. Also, since the TFH and FH chemotherapy regimens were designed to balance overlapping toxicities,^8^, ^9^, ^10^ practitioners using other chemotherapy regimens may expect higher rates of head and neck toxicity that what was demonstrated in this study.

Overall, this study demonstrates the importance of adjuvant CRT in patients with high number of RFs to continue to obtain high rates of locoregional control. Nonetheless, 32% of patients in our series developed distant metastatic disease, which this needs to be addressed. Future prospective evidence is needed to confirm our results and identify which subsets of patients may benefit from adjuvant CRT.

## 5 CONCLUSIONS

Adjuvant CRT, using TFH or FH chemotherapy, for high‐risk salivary gland malignancies is well tolerated. Despite worse features in the adjuvant CRT group, they had equivalent outcomes compared to RT alone. Advanced stage continues to serve as a poor prognostic feature. In addition, the number of RFs should also be considered when contemplating further adjuvant therapy. Since distant metastatic disease represents a primary mode of failure, the number of RFs, serves prognostic significance and should be considered in future prospective trials with specific aims at addressing distant metastatic disease.

## FUNDING

There was no relevant financial support related to this research.

## CONFLICT OF INTEREST

The authors have no conflict of interest to declare.

## TRANSPARENCY STATEMENT

Daniel J. Haraf affirms that the manuscript is an honest, accurate, and transparent account of the study being reported; that no important aspects of the study have been omitted; and that any discrepancies from the study as planned (and, if relevant, registered) have been explained.

## AUTHOR CONTRIBUTIONS

Conceptualization: Benjamin Onderdonk

Data Curation: Benjamin Onderdonk

Formal Analysis: Benjamin Onderdonk

Investigation: Benjamin Onderdonk, Michael Gwede

Methodology: Benjamin Onderdonk, Daniel Haraf

Supervision: Daniel Haraf, Everett Vokes

Visualization: Benjamin Onderdonk

Writing ‐ Original Draft Preparation: Benjamin Onderdonk

Writing ‐ Review and Editing: Benjamin Onderdonk, Everett Vokes, Daniel Haraf, Elizabeth Blair, Nishant Agrawal, Michael Gwede

All authors have read and approved the final version of the manuscript.

Daniel Haraf had full access to all of the data in this study and takes complete responsibility for the integrity of the data and the accuracy of the data analysis.

## Supporting information


**Figure S1** Locoregional Control by Treatment
Kaplan‐Meier locoregional control (LRC) estimates by adjuvant radiation (RT) alone compared to adjuvant chemoradiation (CRT).

**Figure S2 Disease‐free Survival by Treatment**
Kaplan‐Meier disease‐free survival (DFS) estimates by adjuvant radiation (RT) alone compared to adjuvant chemoradiation (CRT).

**Figure S3 Distant Metastasis‐Free Survival by Treatment**
Kaplan‐Meier distant metastasis‐free survival (DMFS) estimates by adjuvant radiation (RT) alone compared to adjuvant chemoradiation (CRT).

**Figure S4 Overall Survival by Treatment**
Kaplan‐Meier overall survival (OS) estimates by adjuvant radiation (RT) compared to adjuvant chemoradiation (CRT).

**Figure S5 Locoregional Control by Stage**
Kaplan‐Meier locoregional control (LRC) estimates by AJCC 7th edition pathologic stage.

**Figure S6 Overall Survival by Lymph Node Positivity**
Kaplan‐Meier overall survival (OS) estimates by lymph node (LN) positivity.

**Figure S7 Overall Survival by Extracapsular Extension**
Kaplan‐Meier overall survival (OS) estimates by extracapsular extension (ECE) positivity.

## Data Availability

The data that support the findings of this study are available from the corresponding author upon reasonable request.
