# Supplementary figures and images for: Adjuvant treatment for high‐risk salivary gland malignancies and prognostic stratification based on a 20‐year single institution experience
Source: Health Sci Rep. 2020 Oct 7;3(4):e195. doi: 10.1002/hsr2.195 (PMC7539565; doi:10.1002/hsr2.195)

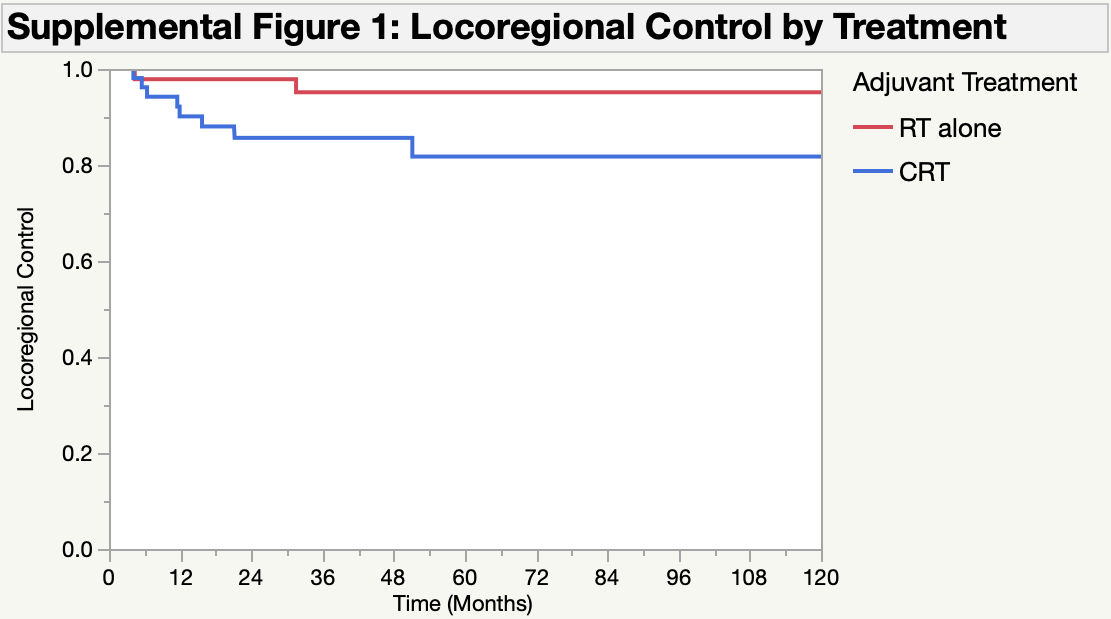

Supplement: Supplementary file 1 — Figure S1 Locoregional Control by Treatment Kaplan‐Meier locoregional control (LRC) estimates by adjuvant radiation (RT) alone compared to adjuvant chemoradiation (CRT). [file HSR2-3-e195-s001.tiff]

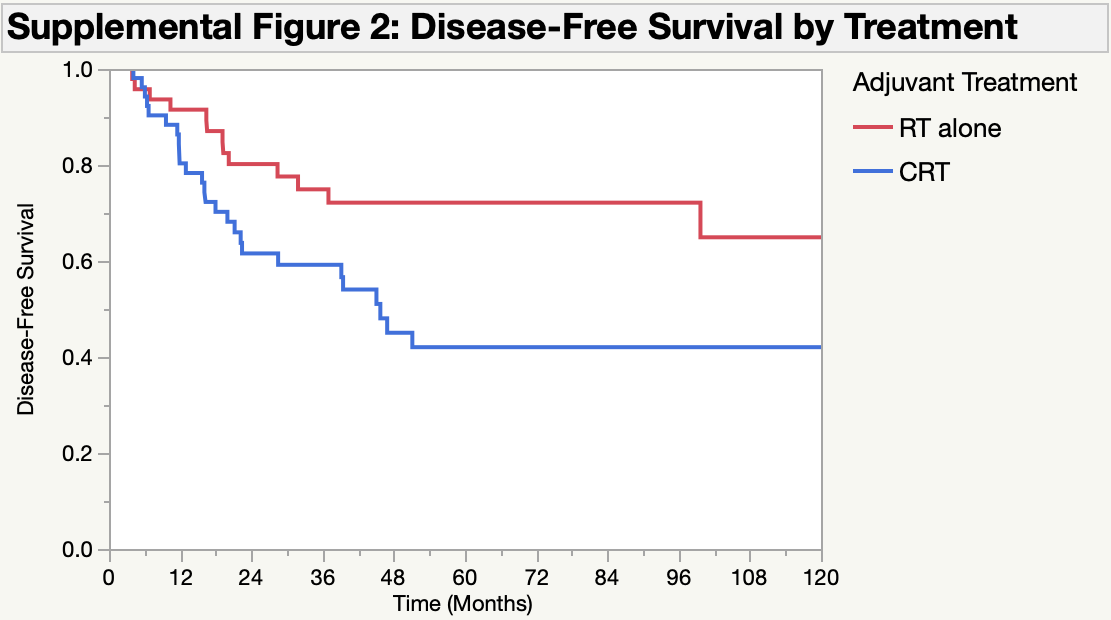

Supplement: Supplementary file 2 — Figure S2 Disease‐free Survival by Treatment Kaplan‐Meier disease‐free survival (DFS) estimates by adjuvant radiation (RT) alone compared to adjuvant chemoradiation (CRT). [file HSR2-3-e195-s002.tiff]

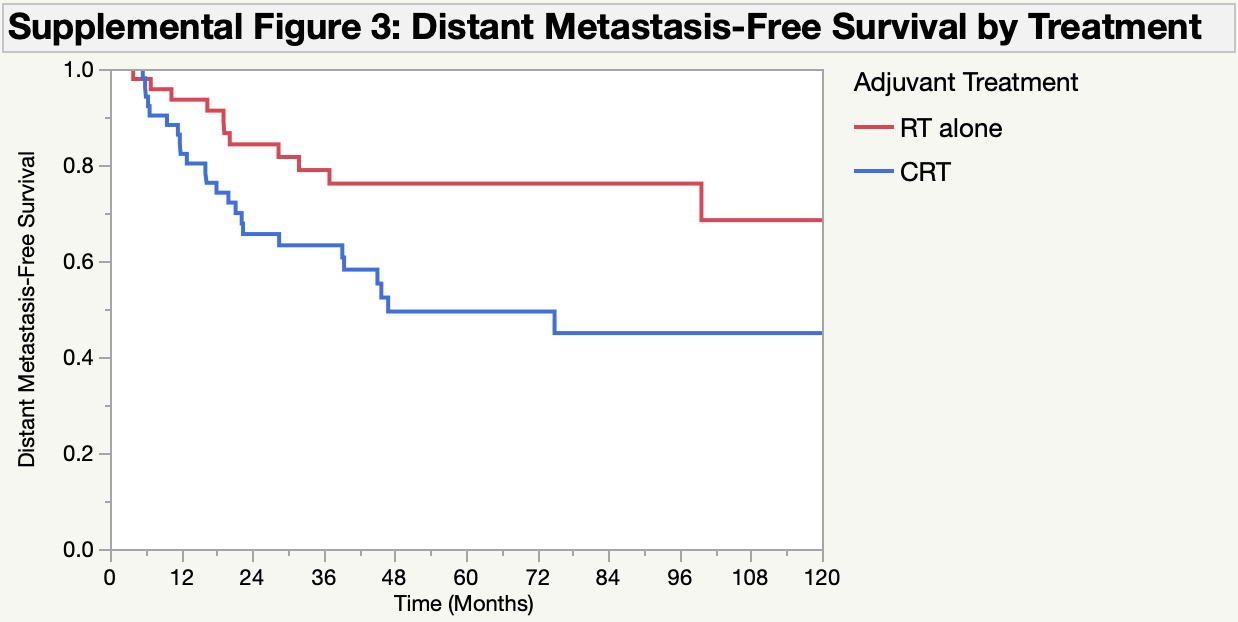

Supplement: Supplementary file 3 — Figure S3 Distant Metastasis‐Free Survival by Treatment Kaplan‐Meier distant metastasis‐free survival (DMFS) estimates by adjuvant radiation (RT) alone compared to adjuvant chemoradiation (CRT). [file HSR2-3-e195-s003.tiff]

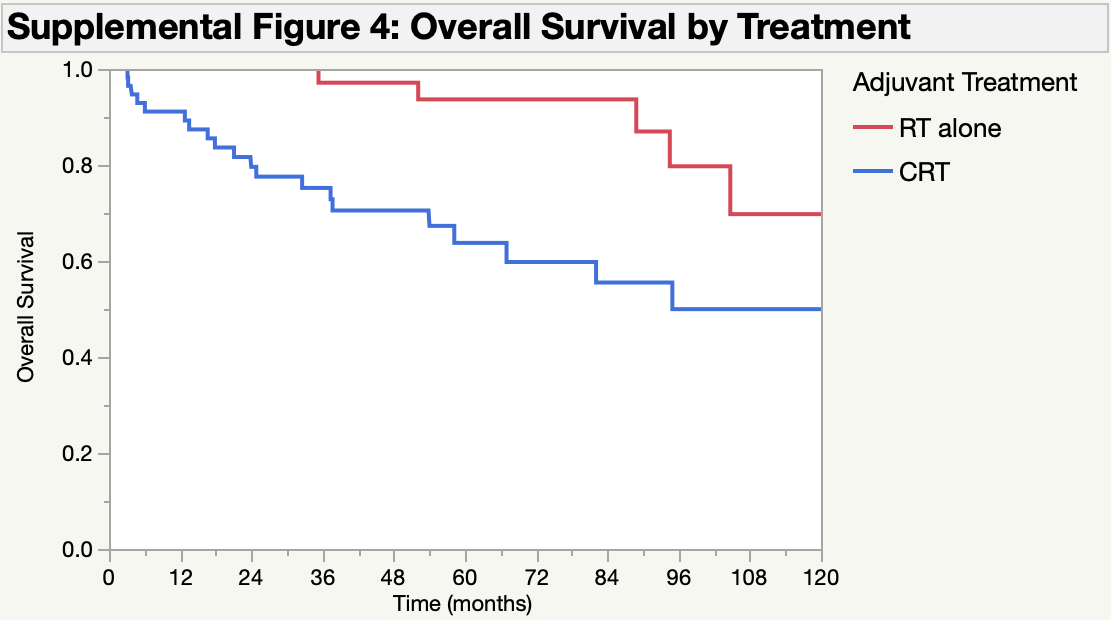

Supplement: Supplementary file 4 — Figure S4 Overall Survival by Treatment Kaplan‐Meier overall survival (OS) estimates by adjuvant radiation (RT) compared to adjuvant chemoradiation (CRT). [file HSR2-3-e195-s004.tiff]

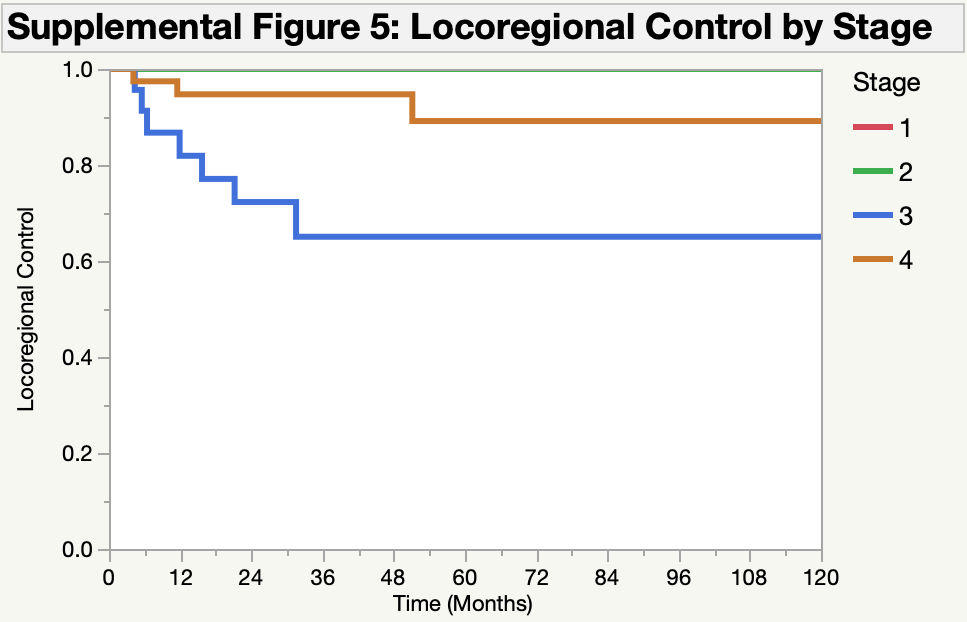

Supplement: Supplementary file 5 — Figure S5 Locoregional Control by Stage Kaplan‐Meier locoregional control (LRC) estimates by AJCC 7th edition pathologic stage. [file HSR2-3-e195-s005.tiff]

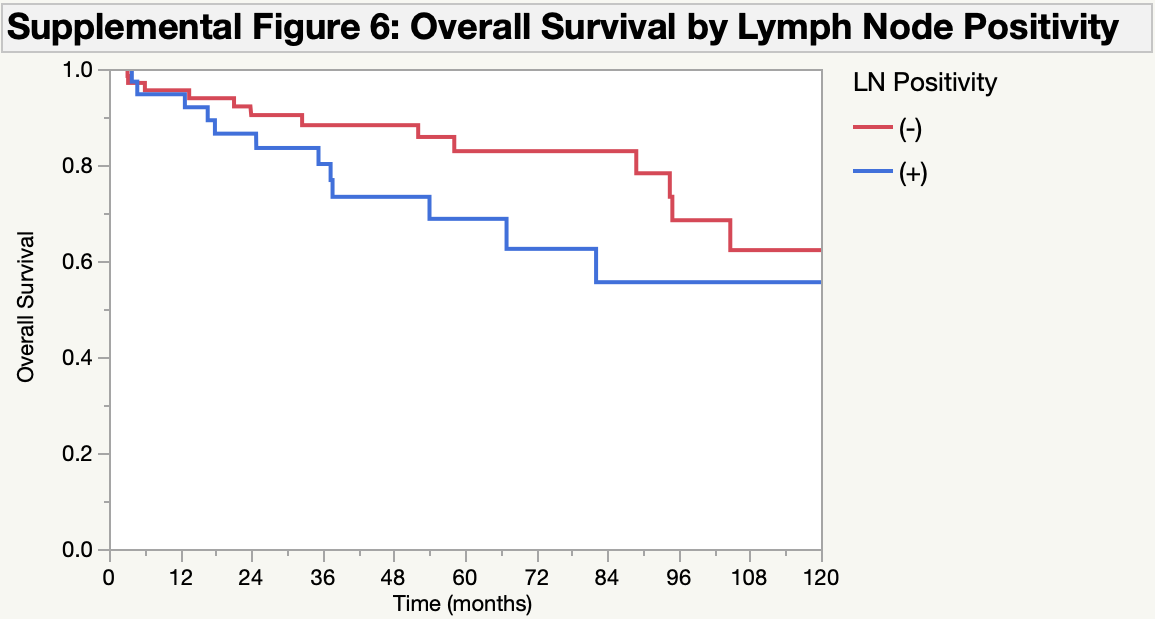

Supplement: Supplementary file 6 — Figure S6 Overall Survival by Lymph Node Positivity Kaplan‐Meier overall survival (OS) estimates by lymph node (LN) positivity. [file HSR2-3-e195-s006.tiff]

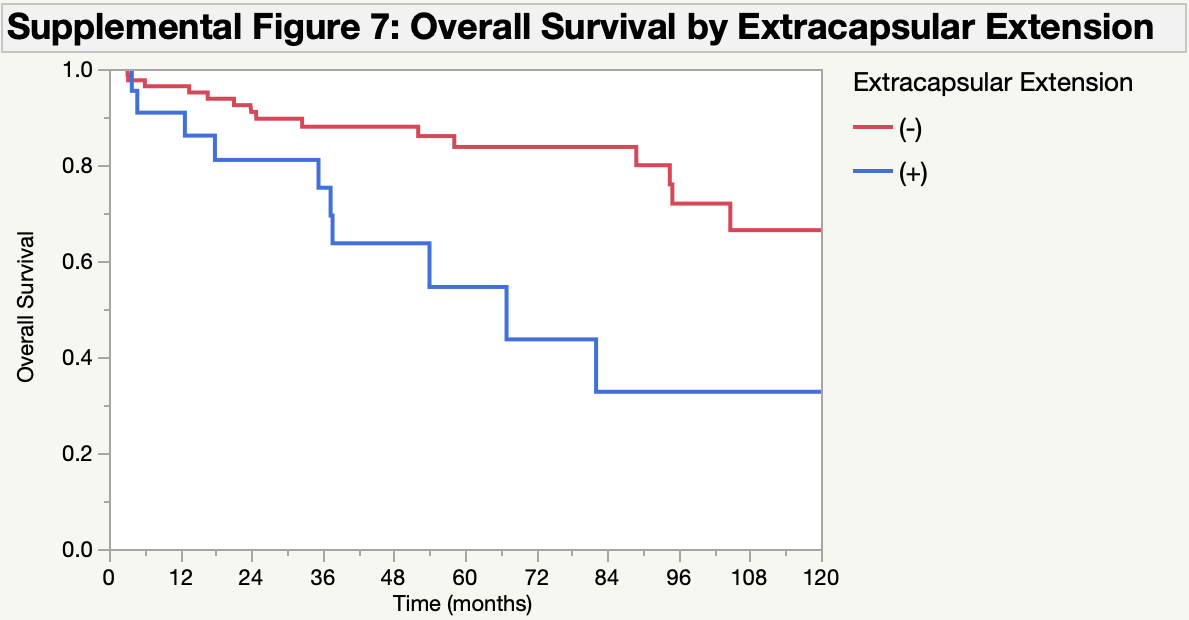

Supplement: Supplementary file 7 — Figure S7 Overall Survival by Extracapsular Extension Kaplan‐Meier overall survival (OS) estimates by extracapsular extension (ECE) positivity. [file HSR2-3-e195-s007.tiff]
